# Digital Twins for Radiopharmaceutical Dosimetry: PBPK Modelling of [^177^Lu]Lu-rhPSMA-10.1 in a Preclinical mCRPC Model

**DOI:** 10.3390/cancers17243957

**Published:** 2025-12-11

**Authors:** Gustavo Costa, Elham Yousefzadeh-Nowshahr, Valentina Vasic, Baiqing Sun, Luca Nagel, Alexander Wurzer, Franz Schilling, Ambros Beer, Wolfgang Weber, Susanne Kossatz, Gerhard Glatting

**Affiliations:** 1 Department of Nuclear Medicine, Ulm University, 89081 Ulm, Germany; gustavo.coelho-alves@uni-ulm.de (G.C.);; 2 Medical Radiation Physics, Department of Nuclear Medicine, Ulm University, 89081 Ulm, Germany; 3 Central Institute for Translational Cancer Research (TranslaTUM), Technical University Munich, 81675 Munich, Germany; baiqing.sun@tum.de (B.S.);; 4 Department of Nuclear Medicine, TUM University Hospital, TUM School of Medicine and Health, Bavarian Cancer Research Center (BZKF), 81675 Munich, Germany

**Keywords:** PBPK modelling, digital twin, [^177^Lu]Lu-rhPSMA-10.1, targeted radionuclide therapy, dosimetry

## Abstract

**Background/Objectives:** Accurate absorbed dose estimation is essential for optimising targeted radionuclide therapy (TRT) in metastatic castration-resistant prostate cancer, where kidney toxicity is dose-limiting. [^177^Lu]Lu-rhPSMA-10.1 is a novel PSMA-targeted radioligand with favourable tumour-to-kidney uptake ratios; however, inter-patient pharmacokinetic variability can lead to differences in organ and tumour absorbed doses under fixed-activity administration. Personalised dosimetry offers a means to address this variability. This work aims to create mouse PBPK model-based digital twins for [^177^Lu]Lu-rhPSMA-10.1 to test the model’s resistance to noise and evaluate its impact on accuracy and absorbed dose calculations. **Methods:** Five CB-17 SCID mice bearing LNCaP tumour xenografts received 2.6–3.1 MBq [^177^Lu]Lu-rhPSMA-10.1 intravenously. Biodistribution was assessed 24 h post-injection by organ weighing and gamma counting. The PBPK model, implemented in MATLAB SimBiology (R2023a), was fitted to individual biodistribution data using mouse-specific physiological parameters. Digital twins—combining the model with fitted parameters—were used to generate time–activity curves (TACs) for kidneys, tumours, and the whole body. Gaussian noise (σ = 0–0.35) was added to TACs to simulate measurement error. The model was refitted, and absorbed doses from time-integrated activities (TIAs) were compared to digital twin references. **Results:** The digital twin approach reproduced experimental data with physiologically plausible parameters. Absorbed dose estimates remained consistent and robust, deviating by <2.3% in kidneys and <1.0% in tumours. **Conclusions:** PBPK-based digital twins enable reliable, individualised dosimetry, even under substantial measurement uncertainty.

## Simple Summary

This paper presents a mouse-specific physiologically based pharmacokinetic (PBPK) model to generate digital twins for dosimetric evaluation of [^177^Lu]Lu-rhPSMA-10.1, a radiopharmaceutical used in targeted therapy for metastatic castration-resistant prostate cancer. Using biodistribution data from five tumour-bearing mice, the authors fit key parameters to create individualized digital twins capable of simulating time–activity curves for organs and tumours. Various levels of Gaussian noise were introduced to mimic measurement uncertainty and to refit the model, assessing its resistance to noise and reliability in estimating time-integrated activities and absorbed doses. Across a wide noise range (σ = 0–35%), absorbed dose estimates for kidneys and tumours remained stable, with mean deviations below 2.3% and 1.0%, respectively, and variability consistently below half of the simulated noise. The study demonstrates that PBPK-based digital twins can provide reliable, individualised dosimetry, even with sparse, noisy data, supporting their potential use to optimise radiopharmaceutical therapy, reduce animal experimentation, and aid translation to personalised human treatment strategies

## 1. Introduction

Prostate cancer is one of the most common cancers worldwide (14.2% [1]), and the typical treatment involves androgen deprivation therapy while the tumours remain responsive. Despite initial responsiveness, the cancer often progresses to metastatic castration-resistant prostate cancer (mCRPC), where the median progression-free survival is approximately 11 months [2]. Prostate-specific membrane antigen (PSMA), a glutamate carboxypeptidase, is frequently overexpressed on mCRPC cell surfaces and can be selectively targeted using radiolabelled ligands that bind to its enzymatic pocket. One of these ligands, PSMA-617, labelled with the beta emitter lutetium-177 (^177^Lu), is approved for targeted radionuclide therapy (TRT) of mCRPC after chemotherapy, having received both Food and Drug Administration (FDA) [3,4] and European Medicines Agency (EMA) approval [5]. In a randomised controlled trial, this TRT, when administered as a fixed activity of 7.4 GBq every six weeks for a total of up to six therapy cycles, improved overall survival by 4 months when added to standard of care therapy (15.3 vs. 11.3 months), which is encouraging, but improvements are still needed [2]. Several other PSMA ligands with improved pharmacokinetics are therefore in clinical development; one of them is the radiohybrid PSMA ligand rhPSMA-10.1 [5,6]. The rhPSMA-10.1 offers the benefit of identical pharmacokinetics, whether used for positron emission tomography (PET) imaging, as with [^18^F]Lu-rhPSMA-10.1, or for therapy, as with [^177^Lu]Lu-rhPSMA-10.1 [7]. Although 85% of mCRPC patients exhibit PSMA uptake, there remains variability in treatment effectiveness [8], as 20% of patients cannot tolerate the standard six cycles of therapy, 50% can tolerate up to ten cycles [9], and the injected activity, in some patients, may be increased by a factor of up to 2.6 [10]. A constant observation among almost all patients is that the kidneys are the dose-limiting organ [9]. Nevertheless, they can still reach a 40 Gy biologically effective dose (BED) without high-grade toxicity [11], highlighting the importance of individualised dosimetry. The use of physiologically based pharmacokinetic (PBPK) models [12,13,14,15] can facilitate a deeper understanding of the pharmacokinetics and biodistribution of radiopharmaceuticals, thereby contributing to ongoing research on these compounds [16]. This approach also allows for testing different therapy regimens, such as prospectively adapting injected activities, the number of cycles, and the interval between them. Likewise, the digital twin—a computational, patient-specific model—integrates imaging data, pharmacokinetic profiles, dosimetric calculations, and physiological parameters to simulate the biodistribution and kinetics of radiopharmaceuticals in an individual patient. This approach enables the prediction of clinically relevant outcomes, including tumour absorbed dose and normal organ toxicity risk, thereby supporting treatment optimisation and personalised strategies in place of fixed, one-size-fits-all administrations [17,18]. A reliable individualisation pipeline may include animal studies, which are a common approach for investigating pharmacokinetics and predicting drug behaviour. A PBPK model can help reduce the number of measurements and experiments, thereby reducing the burden on animals, as noted by Zaid et al. [12].This work aims to create mouse PBPK model-based digital twins for [^177^Lu]Lu-rhPSMA-10.1 to test the model’s resistance to noise and evaluate its impact on accuracy and absorbed dose calculations.

## 2. Materials and Methods

### 2.1. Study Workflow

**Figure 1:**
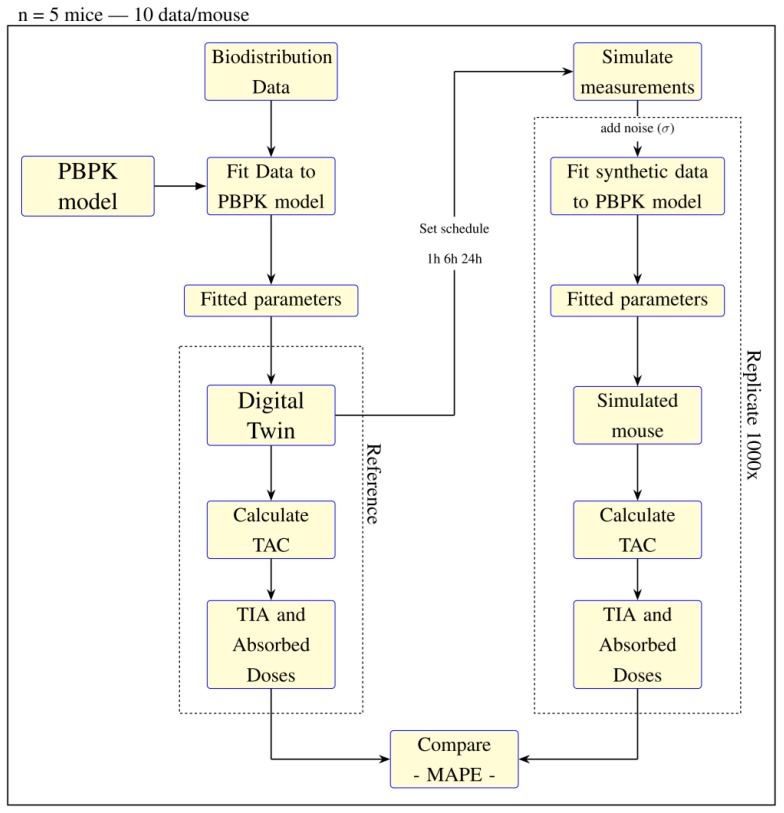
Study’s workflow.

A PBPK model was fitted to the biodistribution data, where six parameters were fitted, generating the digital twin. From the digital twin time activity curves (TACs), time-integrated activity (TIA) and absorbed doses were calculated. An imaging schedule of 1 h, 6 h, and 24 h was set, and the activities were obtained from the digital twin’s TAC. Noise values were added to the activities, and the PBPK model was refitted with five parameters. TAC, TIAs, and absorbed doses were calculated for the simulated mouse, and this process was repeated 1000 times. The simulated mice were compared to the digital twin using MAPE. Figure 1 shows the study workflow.

### 2.2. Experimental Data

The in vivo experiments were conducted in strict accordance with the general animal welfare regulations in Germany and institutional guidelines. Five CB-17 SCID mice aged 6 to 8 weeks bearing LNCap prostate cancer xenografts were studied. Tumour inoculation was performed as described elsewhere [7]. Following isoflurane anaesthesia, 2.6–3.1 MBq (73–87 pmol) of [^177^Lu]Lu-rhPSMA-10.1 was injected into the tail vein. A biodistribution study was conducted 24 h after the injections, when the mice were euthanised, and selected organs were removed, weighed, and their corresponding activities were individually measured in a gamma counter. The dataset has been published previously in [7]. The complete list of measured organs and biodistribution data results can be found in the Supplemental Material Section S1.1 and Table S1, respectively.

### 2.3. PBPK Model

The PBPK model, developed and implemented in SimBiology (MATLAB R2023a), is grounded in well-established physiological and anatomical parameters and has been refined explicitly for TRT and PSMA applications. The model has undergone extensive validation in both humans and mice, and its full description is available elsewhere [12,14,19]. For application in mice, the model was adapted using mouse-specific parameters from the literature [12] and experimental data (Section 2.2). The model incorporates key physiological and physicochemical processes, including blood-flow-driven distribution, diffusion-limited extravasation into interstitial spaces, PSMA-specific binding, receptor-mediated internalisation, intracellular metabolism and release, renal and hepatic clearance, and physical decay of the radionuclide. Tumour lesions and PSMA-expressing organs, including kidneys, liver, and spleen, are explicitly represented, with lesion- and organ-specific receptor densities and blood-flow parameters. Non-PSMA-expressing tissues, such as muscle, adipose tissue, lungs, bone, heart, brain, and skin, are included to capture distribution and clearance accurately. Transcapillary transport is described via the permeability–surface area product and the vascular and interstitial volumes of each tissue, while effective internalisation and release rates capture receptor recycling and intracellular metabolism. The number of receptors (free and occupied) was represented by a single effective value (receptor density), which provides information on PSMA expression and represents the average number of receptors (over time) under the equilibrium of internalisation, recycling, and ligand-receptor-mediated synthesis [15]. Labelled and unlabelled ligands are modelled in parallel systems competing for the same receptor binding, with all physiological parameters assumed to be identical. The dissociation constant and rate, $K_{D}$ and $k_{off}$, were set to 0.43 nmol/L and 0.02 min^−1^, respectively. A whole-body PBPK model integrates population-based physiological parameters, radiopharmaceutical-specific characteristics, and individual data; for a new radiopharmaceutical, relevant parameters must be adapted and, when required, the compartmental structure refined. This framework was applied to [^177^Lu]Lu-rhPSMA-10.1, using the backbone of the original model developed for [^68^Ga]Ga-PSMA-11. The full list of organs included in the model is provided in the Supplemental Material Section S1.3.

### 2.4. Mouse Digital Twin

The digital twins were created by fitting six key parameters, among the most important parameters that drive the absorbed dose (AD) variability [13], to the biodistribution study. These parameters are receptor densities in the tumour, kidneys, liver, and spleen; blood flow to the tumour; and glomerular filtration rate (GFR). The PBPK model was fitted to the biodistribution data of ten selected organs (Supplemental Material Section S1.1), which consists of the activity of these organs at 24 h. Thus, the method involves fitting ten data points into a single, complex function. Moreover, the use of a single 24 h time point is supported by the model’s widespread application in humans, which has consistently produced robust results. In addition, injected activity and individual organ weights were included. The fitting process was performed in SimBiology (MATLAB R2023a), and the estimated parameters were used as starting values for the next round of fitting. Thus, the mouse digital twin corresponds to the PBPK model combined with the individually fitted parameters—such as receptor densities, blood flow, and GFR—derived from subject-specific measurements.

### 2.5. Simulations

From a typical clinical schedule of 4 h, 24 h, 72 h, and 7 d [5,20], and assuming a human-to-mouse time scaling factor of 7 [21], a measurement schedule of 1 h, 6 h, and 24 h was defined to obtain reference (in silico) activities in the kidneys, tumour, liver, and the total body (TB) from the TACs of the digital twins. This schedule accommodates animal welfare, working hours, and project-specific restrictions, such as a maximum of two measurements per day and a minimum of two hours between measurements. Furthermore, it mirrors ongoing experimental protocols in our group, ensuring coherence between simulated and forthcoming empirical datasets. The PBPK model was refitted to noise-added activities (Equation (1) [22]), with seven Gaussian noise levels (σ), and the fitting procedure was repeated j = 1000 times per σ.

(1) $$A_{sim,j}=A_{ref}+A_{ref}\;\times \;g\left(\mu ,\sigma \right)$$

where $A_{sim,j}$ is the simulated activity in the organ or tumour at the j^th^ replication, $A_{ref}$ is the reference value from the digital twin, and the random value g(μ,σ) is taken from the Gaussian noise function (using the Matlab “NORMRND” function), where the mean μ = 0 and σ∈{0.05,0.10,0.15,0.20,0.25,0.30,0.35}. The σ values were chosen to identify the linear response from the model and to comprise errors found in single-photon emission computerised tomography (SPECT) quantification [23].

Model fitting was performed for five PBPK model parameters: receptor densities in the liver, tumour, and kidneys; glomerular filtration rate (GFR); and blood flow to the tumour. Due to parameter non-identifiability, the receptor density in the spleen was fixed to the fitted value (Table 1) for each individual mouse. For each replicate, TACs were generated, from which the relative deviation of the TIAs from the reference value—denoted as the delta TIA (ΔTIA)—was computed as follows:

(2) $$\Delta TIA=\frac{TIA_{sigma,j}-TIA_{DT}}{TIA_{DT}}$$

where $TIA_{sigma,j}$ is the TIA with added σ at the j^th^ replication, and $TIA_{DT}$ is the TIA of the digital twin. Additionally, the mean percentage absolute error (MAPE) was determined:

(3) $$MAPE=\frac{1}{N}\times \sum_{n=1}^{N}\Delta TIA$$

where N=1000 is the number of replications.

This work relies on two statistical measures: the mean and the median. The mean is calculated based on 1000 replications; with this large sample size, random variations and outliers tend to balance each other out, making the mean a suitable measure. On the other hand, the median is calculated from a smaller sample size (n = 5), which is appropriate because a single outlier or extreme value can significantly distort the mean, making it an unreliable representation of the typical observation.

### 2.6. Absorbed Dose

The absorbed dose is calculated by the following Equation (4):

(4) $$D=\frac{E}{m}$$

where E is the energy deposited in the volume of mass m, and D is the absorbed dose. The deposited energy was calculated from the total number of disintegrations, which is the area under the curve (AUC) of the TAC, from 0 to 124 h, and the energies of the beta-particle emissions, along with their corresponding emission probabilities (Supplemental Material Table S2). The reference absorbed doses were calculated from the TACs of the digital twins, which were used analogously as a reference for the MAPE (Equation (3)). The AD was calculated based on self-irradiation and local deposition, as energy deposition by β-particles, in a 10 mm sphere, accounts for 90% [24], with a maximum range of 1.8 mm [25,26].

## 3. Results

The mean values of the fitted parameters for the digital twins (Section 2.4) are presented in Table 1. Blood flow to the tumours varied more markedly than other parameters, which is likely attributed to differences in tumour size (range of 39 mg to 109 mg) and tumour heterogeneities.

**Table 1:** Fitted parameters.

Parameter	m1	m2	m3	m4	m5	Median	Units
RD_Tum	18.21	19.52	20.00	20.56	17.45	19.52	nmol/L
F_Tum	3.52 × 10 ^−6^	5.41 × 10 ^−6^	1.82 × 10 ^−5^	5.61 × 10 ^−6^	3.72 × 10 ^−6^	5.41 × 10 ^−6^	L/min
RD_Kid	4.92	4.77	8.35	6.78	11.14	6.78	nmol/L
GFR	3.54 × 10 ^−5^	3.35 × 10 ^−5^	3.71 × 10 ^−5^	3.68 × 10 ^−5^	3.41 × 10 ^−5^	3.54 × 10 ^−5^	L/min
RD_Liv	0.31	0.28	0.60	0.31	0.26	0.31	nmol/L
RD_Spl	0.40	0.25	0.28	0.31	0.39	0.31	nmol/L

RD_Tum, RD_Kid, RD_Liv, and RD_Spl are the receptor densities in the tumour, kidneys, liver, and spleen, respectively. F_Tum is the blood flow to the tumour.

**Figure 2:**
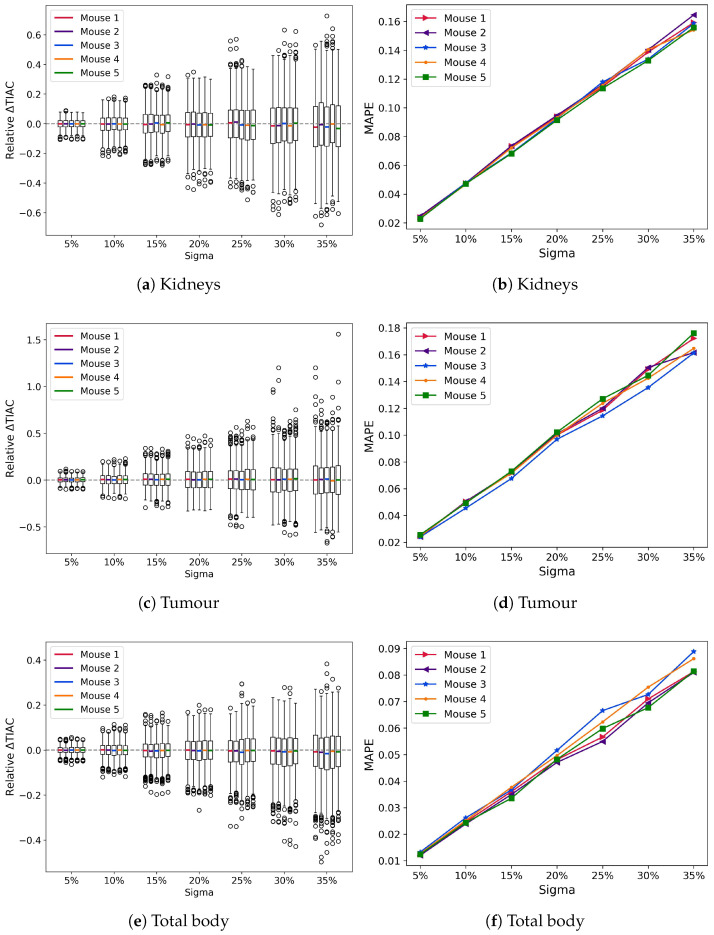
Box plots of the relative TIA difference for all mice DTs in the kidneys (**a**), tumour (**c**), and TB (**e**), where the median values show small deviations from zero and the interquartile ranges increase linearly with the added error; and MAPE of TIA for all mice in the kidneys (**b**), the tumour (**d**), and in TB (**f**). The MAPE increases linearly with σ for all mice with deviations of less than half the added noise, throughout all tested σ values.

A comparison of 140,000 TACs revealed that the differences in the median values of the TIAs across all tested σ-values and the reference value were close to zero, with deviations in the median of less than 5% (Figure 2); however, the interquartile ranges and whiskers increased with noise. There is a slight tendency to underestimate TIAs in TB at σ = 35%; in contrast, this trend is less significant in the kidneys and is not observed in the tumour (Figure 2a,c).

**Figure 3:**
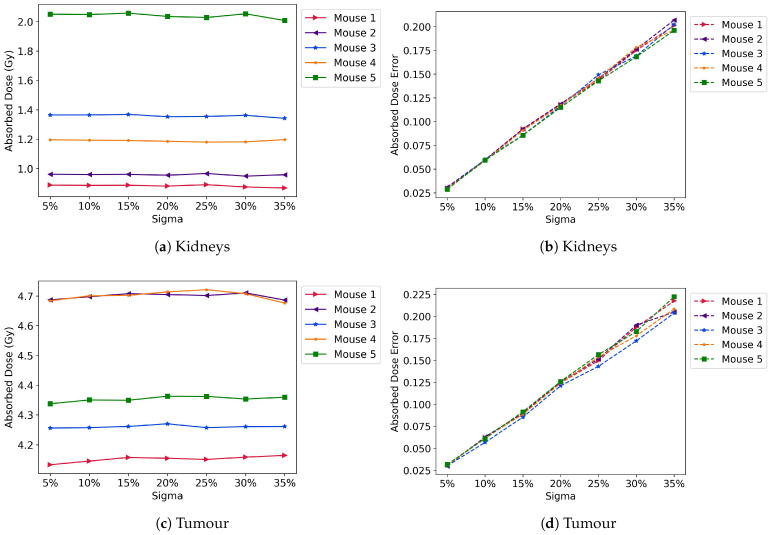
Absorbed doses for all mice in the kidneys (**a**) and tumour (**c**), and their corresponding errors (**b**) and (**d**), respectively. The deviation is less than half the added noise throughout all tested σ.

The MAPE increased linearly with the added noise (σ) for both the kidneys and the tumour (Figure 2a,c). Greater variability was observed for σ values of 30% and 35% in the tumour than in lower sigma values; however, in all cases, the MAPE remained lower than 50% of the σ. The tumour (Figure 2d) exhibited a slightly larger variation between mice compared to the kidneys, likely due to the challenges in predicting blood flow to the tumour and the significant variability in tumour sizes and receptor expression. The kidney absorbed doses (mean ± SD) for mice 1 to 5 were 0.88 ± 0.10, 0.95 ± 0.11, 1.35 ± 0.15, 1.18 ± 0.13, and 2.03 ± 0.23, respectively (Figure 3a). As expected, the standard deviation increased linearly with sigma (Figure 3b). The ADs reported here are for σ = 20% as it is the median value; the ADs for all noise values are presented in the Supplemental Material (Table S3).

Similarly to the kidneys, the ADs to the tumour remained close to a constant value across all noise realisations (Figure 3c), and the standard deviation increased linearly with sigma, with larger differences for σ = 30% and σ = 35% (Figure 3d). In all cases, the standard deviation of ADs is less than half of the σ value. The mean absorbed doses in the tumour and kidneys were accurately predicted with all tested σ, with maximum discrepancies of the mean values to the reference values of 0.96% and 2.32%, respectively (Table 2).

**Figure 4:**
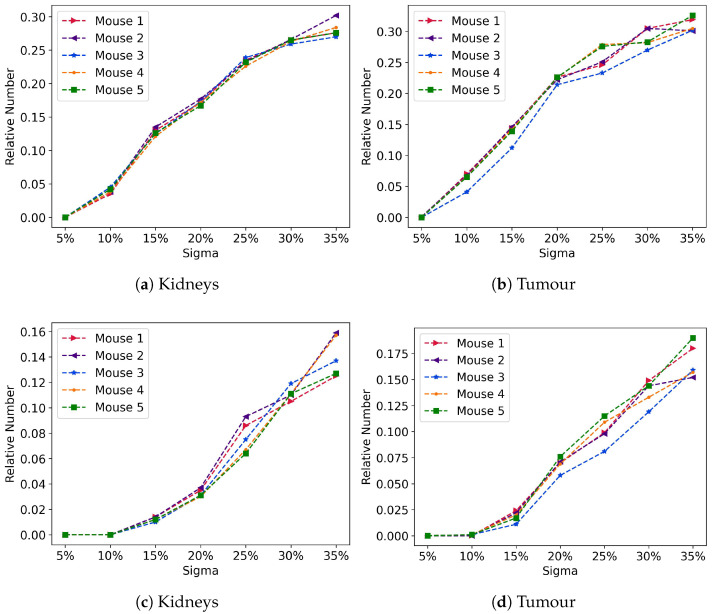
Relative number of AD over 10% of the reference value for the kidneys (**a**) and tumour (**b**), and relative number of AD over 20% for the kidneys (**c**) and tumour (**d**).

There are no replications with AD deviation over 10% from the reference value at σ = 5% for either the tumour or the kidneys (Figure 4a,b). Nonetheless, the number of replications that showed deviations over 10% increased linearly for both the tumour and kidneys, reaching values similar to each respective σ. In addition, there is a decrease in the slope from σ = 25% in the tumour. The number of replications with AD deviation over 20% was smaller for both the kidneys and tumour, with no AD over 20% for σ = 5% and σ = 10%, and maximum relative value below 18% for the tumour and 16% for the kidneys at σ = 35% (Figure 4c,d).

## 4. Discussion

This work aimed to provide mouse PBPK model-based digital twins for [^177^Lu]Lu-rhPSMA-10.1 and to evaluate the effect of added noise on accuracy and absorbed dose calculations. Here, the digital twin is composed of the model itself along with six fitted parameters and individual (measured) parameters, which are used as reference values. During the replication, five of the six parameters are fitted to activities taken from the TAC (with noises of 0–35%), and those newly fitted parameters are used to simulate the pharmacokinetics, which will generate new TACs for new TIAs. The new TIAs are compared to the reference value to verify how far off they are. Our study demonstrated that a PBPK-based digital twin approach, even with significant noise, is capable of predicting the TIA with accuracy within 50% of the simulated measurement noise and can accurately estimate AD for both tumours and kidneys. Notably, the AD variability due to noise was under 50% of the added error for the investigated schedule, reinforcing the robustness of this computational approach. The mean AD deviation from the reference value remained below 2.3% for kidneys and 1.0% for tumours and was consistent across the wide range of investigated noise (σ = 0–35%). It is worth noting that the estimated accuracy depends on the replications, and its comparison with the reference value was possible only because the organs’ weight and activity could be reliably measured.

The precision of the AD can vary by up to 30% as much as 20% of the time, along with measurement errors. This is particularly relevant, as clinical and preclinical dosimetry data often suffer from variability due to limited imaging time points and biological differences among subjects. However, precision can be increased within a Bayesian framework, utilising population data, which will be incorporated in future steps. As shown in earlier studies, human kidneys can tolerate BEDs of up to 40 Gy without high-grade toxicity. Our findings align with reports highlighting the critical role of patient-specific dosimetry in identifying the “therapeutic window”, thereby improving therapy outcomes while avoiding overly conservative absorbed-dose limits. For instance, the European Association of Nuclear Medicine (EANM) recommends incorporating individualised dosimetry to better estimate the therapeutic window between destroying cancer tissue and preserving healthy tissues, ultimately preventing undertreatment [16], which is a common issue for many patients receiving radiopharmaceutical therapy [27]. Moreover, the digital twin methodology enables the simulation of diverse therapeutic scenarios—including variations in labelled/unlabelled ligand ratio, activity, and cycle frequency—without an additional experimental burden. By fitting organ-specific receptor densities and physiological parameters, our model captures heterogeneity in radioligand uptake and clearance, which is essential for accurate TIA and AD computation. These fitted receptor densities showed biologically plausible values and were comparable to those reported in human studies (e.g., 18 nM in kidneys and 46 nM in tumours) [14], with our mouse model reflecting approximately half those values, 7 nM and 19 nM, respectively, consistent with species-specific PSMA expression differences [28,29]. It is essential to emphasise that any animal model is, at best, an approximation of the human mechanism; in contrast, it can provide relevant information on pharmacokinetics, biodistribution, and dosimetry, despite variations between animals and humans that occur when translation is performed [16]. Therefore, there are crucial differences that must be considered when translating between species. For instance, PSMA is not expressed by the mouse prostate [28]. Therefore, no PSMA receptors were included in the prostate, and the activity in the prostate was related to its presence in the vascular and interstitial spaces. Similarly, Roy et al. [29] reported decreased PSMA expression in mouse salivary glands, and that a toxicity study may even be unreliable. In our study, fitting the model to the parotid and submandibular glands resulted in a low and near-zero value, indicating an equally good fit when the number of receptors was set to zero. In addition, during the replication phase, the receptor density in the spleen was fixed to the value of each corresponding digital twin, as several different values can result in an equally good fit, known as a non-identifiable parameter issue [30,31].

Most PBPK model parameters were within the physiological range, with fixed values taken from the literature and experimental data. This approach enhances biological plausibility and reduces uncertainty in model predictions. Nevertheless, there is a limitation to using a single time point to generate the digital twin, but ongoing studies address this by incorporating earlier time points alongside the biodistribution data. The single time point is not considered critical, even though it may contribute to GFR fitting. The mean GFR of 1.77 μL/min/g is lower than values reported in previous studies (8–12 μL/min/g [32,33]). This difference can likely be explained, in addition to the mentioned limitation, by (1) differences in mouse strains and (2) possible nephropathy, as it aligns with findings by Sasaki et al., where induced nephropathy decreased GFR to 2.23 μL/min/g. This work is also limited by the use of mouse models, which inherently have species-specific differences, particularly in biodistribution, such as the lack of uptake in the salivary glands and prostate, which would make the xenograft model incompatible if a study on xerostomy were involved. In addition, while this study is limited by a small sample size (n = 5) and a larger cohort would allow for a more extensive biological analysis, the primary objective of this study was to evaluate the model’s resistance to noise. Once the digital twin is constructed, noise propagation is governed by the model rather than sample size. Translation to human models will be addressed in future work, in which mouse-specific parameters will be replaced with human-specific values and the model will be expanded to incorporate multiple tumours, as well as PSMA expression in salivary glands and the prostate.

The presented approach enables the fine-tuning of therapeutic regimens based on individual biodistribution profiles through non-invasive simulation of different dosing scenarios, which allows for the optimisation of injected activity and treatment cycles while minimising toxicity to organs at risk. Furthermore, the methodology reduces the need for extensive animal experimentation by maximising the information extracted from minimal data, aligning with ethical principles and animal welfare regulations. Finally, this study highlights the potential for PBPK models to serve as a translational tool for human applications, provided that appropriate physiological scaling is applied. This could help bridge the gap between preclinical findings and clinical implementation, supporting a personalised dosimetry-driven approach to PSMA-targeted therapies and treatment planning.

## 5. Conclusions

This work demonstrates the feasibility of a PBPK model-based mouse digital twin framework for calculating absorbed doses in mCRPC therapy. It is important to note that the focus of this work was on presenting a PBPK model-based dosimetry and digital twins, rather than on the exact precision of the absorbed dose or how closely the model replicates individual animals. Using batch computation was essential for accurate AD determination, with replication and analysis requiring less than two hours of computational time. Even with substantial simulated noise (up to σ = 35%), deviations from the reference value remained below the simulated noise level while effectively capturing inter-individual variability in receptor density, supporting therapy individualisation. With appropriate physiological scaling, PBPK model-based digital twins offer strong translational potential for PSMA-targeted radiopharmaceutical therapy. Their integration into a treatment workflow can enhance dosimetry predictions, optimise treatment cycles and injected activities, and improve safety, while reducing the need for extensive animal experimentation and aligning with the principles of personalised therapy. Next steps focus on expanding this work’s approach by using ongoing experiments that include a three-time-point image-based TAC, a 24 h biodistribution study, and pre-therapeutic PET data.

## Figures and Tables

**Table 2:** Maximum discrepancy of the mean absorbed dose in the tumour and kidneys when compared to the reference value. Maximum discrepancy across all tested σ.

	Difference (%)	
	**Kidneys**	**Tumour**
Mouse 1	2.32	0.96
Mouse 2	0.38	0.15
Mouse 3	1.77	0.12
Mouse 4	0.06	0.02
Mouse 5	2.20	0.83

## Data Availability

The original contributions presented in this study are included in the article/Supplementary Material. Further inquiries can be directed to the corresponding author.

## Supplementary Materials

The following supporting information can be downloaded at: https://www.mdpi.com/article/10.3390/cancers17243957/s1, Table S1. Biodistribution of [^177^Lu]Lu-rhPSMA-10.1, at 24 h p.i. in male LNCaP tumor-bearing SCID mice. Data are expressed as a percentage of the injected dose per gram (% ID/g), mean ± standard deviation (SD; n = 5). Table S2. Lu-177 beta transitions and their corresponding probabilities. Table S3. Mean absorbed doses (Gy) and standard deviation (Gy) for the tumour and kidneys for all σ.
