# Supplementary material for: Digital Twins for Radiopharmaceutical Dosimetry: PBPK Modelling of [177Lu]Lu-rhPSMA-10.1 in a Preclinical mCRPC Model
Source: Cancers (Basel). 2025 Dec 11;17(24):3957. doi: 10.3390/cancers17243957 (PMC12730921; doi:10.3390/cancers17243957)
Supplement: Supplementary file 1 [file cancers-17-03957-s001.zip › cancers-3969918-supplementary.pdf]

Supplemental Material

S1.1 List of measured organs/tissues

The biodistribution study included weight and activity measurements of the following: blood, heart, lungs, liver, spleen, pancreas, stomach, intestine, kidney, muscle, bone, tail, tumor, parotid gland, and submandibular gland.

S1.2 Biodistribution of [^{177}Lu]Lu-rhPSMA-10.1

Table S1: Biodistribution of [^{177}Lu]Lu-rhPSMA-10.1, at 24 h p.i. in male LNCaP tumor-bearing SCID mice. Data are expressed as a percentage of the injected dose per gram (% ID/g), mean ± standard deviation (SD; n=5).

Uptake in %ID/g	mean	SD
blood	0.0009	0.0001
heart	0.017	0.001
lung	0.032	0.004
liver	0.18	0.06
spleen	0.17	0.03
pancreas	0.013	0.002
stomach	0.056	0.014
intestine	0.11	0.05
kidney	1.97	0.78
adrenals	0.06	0.04
muscle	0.003	0.002
bone	0.022	0.008
tumor	9.82	0.3
parotid gl.	0.041	0.009
submand. gl.	0.037	0.007

S1.3 List of modelled organs/tissues

The PBPK model included the following tissues: skin, adipose tissue, bones, red bone marrow, brain, mucle, heart, lungs, lacrimal gland, parotif gland submandibular gland, prostat, gastrointestinal tract, liver, spleen, kidneys, and remainder of the body.

S1.4 Lu-177 beta decay

Table S2: Lu-177 beta transitions and their corresponding probabilities

Energy (keV)	Probability (%)
177.0	11.64
248.6	0.012
385.4	9.1
498.3	79.3

S1.5 Absorbed dose calculation

$$D = \sum_{n=1}^{4} \frac{TIAC \times beta_i \times P_i \times keVToJoules \times F}{m} \tag{Eq. S1}$$

Where, $beta_i$ is the ith beta energy (table S2), P_i is the corresponding beta emission probability, F = $10^6 \times 3600$ is the conversion factor from MBq.h to Bq.s, keVToJoules = 1.6×10^{-16}, is the energy conversion from keV to J, TIAC = time integrated activity coefficient [MBq.h], and m = mass of the volume [kg]

Table S3: Mean absorbed doses (Gy) and standard deviation (Gy) for the tumour and kidneys for all σ.

	$\sigma = 5\%$		$\sigma = 10\%$		$\sigma = 15\%$		$\sigma = 20\%$		$\sigma = 25\%$		$\sigma = 30\%$		$\sigma = 35\%$	
m1	0.89	± 0.03	0.88	± 0.05	0.89	± 0.08	0.88	± 0.10	0.89	± 0.13	0.87	± 0.15	0.87	± 0.17
	4.13	± 0.13	4.15	± 0.26	4.16	± 0.37	4.15	± 0.52	4.15	± 0.62	4.16	± 0.78	4.16	± 0.91
m2	0.96	± 0.03	0.96	± 0.06	0.96	± 0.09	0.95	± 0.11	0.97	± 0.14	0.95	± 0.17	0.96	± 0.20
	4.69	± 0.14	4.70	± 0.29	4.71	± 0.42	4.70	± 0.59	4.70	± 0.71	4.71	± 0.89	4.69	± 0.96
m3	1.36	± 0.04	1.36	± 0.08	1.37	± 0.12	1.35	± 0.16	1.35	± 0.20	1.36	± 0.23	1.34	± 0.27
	4.26	± 0.13	4.26	± 0.24	4.26	± 0.36	4.27	± 0.52	4.26	± 0.61	4.26	± 0.73	4.26	± 0.87
m4	1.20	± 0.04	1.19	± 0.07	1.19	± 0.11	1.18	± 0.14	1.18	± 0.17	1.18	± 0.21	1.20	± 0.23
	4.68	± 0.14	4.70	± 0.29	4.70	± 0.42	4.71	± 0.58	4.72	± 0.73	4.71	± 0.84	4.68	± 0.97
m5	2.05	± 0.06	2.05	± 0.12	2.06	± 0.18	2.03	± 0.23	2.03	± 0.29	2.05	± 0.35	2.01	± 0.39
	4.34	± 0.14	4.35	± 0.27	4.35	± 0.40	4.36	± 0.55	4.36	± 0.68	4.35	± 0.80	4.36	± 0.97
